# Transient Early Mechanical Loading Induces Hypertrophic Chondrocyte Differentiation of Human Mesenchymal Stromal Cells

**DOI:** 10.3390/cells14221773

**Published:** 2025-11-12

**Authors:** Sina Enzmann, Aline N. Klaus, Romano Matthys, Esther Wehrle, Martin J. Stoddart, Sophie Verrier

**Affiliations:** 1 AO Research Institute Davos, 7270 Davos Platz, Switzerland; 2 RISystem AG, 7302 Landquart, Switzerland

**Keywords:** human MSC, mechanical strain, hypertrophic chondrocytes, in vitro, 3D, bioreactor, bone-healing

## Abstract

**Highlights:**

**What are the main findings?**

- Mechanical stimulation induces hypertrophic differentiation of naïve MSCs.
- Short early stimulation is as efficient as prolonged stimulation.

**What is the implication of the main finding?**

- Our findings could guide future in vivo studies exploring the effect of mechanical stimulation on healing outcomes.
- Our data could provide in vitro support for the development of smart implants.

**Abstract:**

Optimal mechanical parameters for successful bone-healing remain unclear despite their critical influence on fracture outcomes, and the timing of post-surgery mobilization is still controversial despite many clinical observations and pre-clinical studies. In this bioreactor in vitro work, we investigate the effect of fundamental parameters such as timing, duration, and frequency of mechanical stimulation on the endochondral bone-healing paths, specifically on the hypertrophic chondrocyte differentiation of naïve human mesenchymal stromal cells (hMSCs). Human MSCs encapsulated in Gelatin-Methacryloyl hydrogels (GelMa) were subjected to three different 10% strain protocols: P1 (168 long-break cycles spread over 14 days), P2ce (cycle equivalent: 168 short-break cycles condensed in 42-min stimulation followed by 14 days free swelling), and P2te (time equivalent—14 days continuous stimulation, 80′640 short-break cycles). In the free-swelling control group, samples were cultured for 14 days without any mechanical stimulation. Our results confirmed that 10% strain induces a robust hypertrophic chondrocyte differentiation of naïve MSCs in all three tested protocols, as demonstrated by enlarged cell size, rounded morphology, robust upregulation of hypertrophic markers (*COL10A1*, *MMP13*, *RUNX2*, *ALP*), and reduced glycosaminoglycan production. Of particular interest, we show that P2ce (early short stimulation) was as effective as the two extended stimulation protocols, suggesting that initial mechanical signals are sufficient to trigger cell differentiation toward a hypertrophic chondrocyte phenotype that continues even after stimulation ceases. These in vitro findings provide crucial insights into the cellular basis of endochondral ossification during the early phase of loading and show a beneficial long-term effect of early mechanical stimulation. By demonstrating that the cellular mechanobiology of hypertrophic differentiation responds to brief early stimulation, our findings provide a scientific foundation to guide future in vivo investigations on how rehabilitation protocols could influence fracture healing.

## 1. Introduction

The success of bone-healing highly depends on the mechanical environment at the fracture site. In clinical and pre-clinical setups, physical and biological aspects of a fracture will influence its outcome [1,2], with both the size and stability of a fracture impacting the healing route [3]. While primary bone-healing requires absolute stability, secondary bone-healing is initiated by the presence of motion at the fracture site [4]. Perren and Courday [5] developed the strain theory, in which they describe the influence of the amount of motion related to the size of the fracture gap as a percentage of strain. At the biological level, mechanical stimulation promotes the formation of cartilaginous callus, aiming to reduce fracture motion for further bone-healing, and a clear relation between motion and callus size has been shown [6,7,8,9,10]. Numerous studies have since investigated the role of stability on bone fracture healing in pre-clinical or clinical investigations, leading to the design and/or choice of appropriate implants or rehabilitation protocols [11,12,13]. For example, cyclic compression applied with optimal time, rate, and magnitude across the fracture site was shown to accelerate and enhance callus formation [3,11,12,14,15,16]. While the incidence of interfragmentary motion on callus formation is widely accepted, to date, there are no guidelines in clinical applications on how much, when, and for how long mechanical stimulation should be allowed or promoted [8,17,18].

From a cellular point of view, the mechanical environment has a direct effect on the locally recruited mesenchymal stromal cells (MSCs) [19,20,21,22,23,24]. Absolute stability was shown to promote direct (haversian) bone-healing, in which MSCs differentiate towards osteoblasts, directly depositing bone matrix, hence healing bone. On the other hand, during endochondral bone-healing, ossification proceeds through a sequence of events including the formation of a hematomata, recruitment and differentiation of MSCs, and the formation of a transient cartilaginous callus that will gradually remodel into bone tissue [6]. During endochondral ossification, mechanical instability/stimulation drives MSCs differentiation towards an intermediate hypertrophic chondrocyte phenotype and associated cartilaginous matrix that will eventually remodel and undergo ossification. At the cellular level, questions remain about the optimal deformation conditions and timing for the effective healing process. The necessity, the magnitude, and the conditions of early mechanical stimulation are still debated clinically and scientifically.

In a recent in vitro study using a uniaxial loading multi-well bioreactor [25], we investigated the effect of different percentages of strain on the hypertrophic chondrocyte differentiation of naïve MSCs. Our results indicated that the continuous application of 10% strain cyclic deformation over 14 days induces a hypertrophic phenotype of hydrogel-embedded naïve MSCs. In this study, we investigate how both the number of deformation cycles, the length of the inter-cycle break, and the overall duration of the deformation period affect MSCs differentiation. We hypothesize that brief early stimulation with 10% strain is as effective as longer protocols in promoting hypertrophic differentiation of naïve MSCs.

## 2. Materials and Methods

### 2.1. Cellularized Hydrogel Preparation

#### 2.1.1. Cell Isolation, Expansion

Human bone marrow isolated MSCs from 6 anonymized donors were used in this study (female, 18 to 73 years old, average 53 ± 21.5). Patients’ general consent was obtained in accordance with the Swiss Human Research Act, permitting the use of anonymized biological material and associated health-related data for research purposes (see paragraph Institutional Review Board Statement and Informed Consent Statement). After standard density gradient separation (Histopaque-1077, Sigma, Buchs, Switzerland), MSCs were sub-cultured (3 × 10^5^ cells/cm^2^) in αMEM (Gibco, Life Technology, Reinach, Switzerland) containing 10% FCS (Corning, Milian, Vernier, Switzerland) and 5 ng/mL bFGF (Fitzgerald, Bray, WI, Ireland) in a 37 °C-5% CO_2_ incubator. Cell-culture medium was changed twice a week, and cells were sub-cultured at 80% confluence. Cells of passage 2 to 4 were used.

#### 2.1.2. Hydrogel Preparation

Gelatin-Methacryloyl (GelMa) hydrogels were synthesized from porcine gelatine (Sigma) as described in [26]. Briefly, a 10% (*w*/*v*) gelatine solution was prepared in PBS and maintained at 60 °C under constant stirring, while 1.4 mL of methacrylic anhydride (Sigma–Aldrich, Buchs, Switzerland) was added dropwise. The reaction mixture was then incubated at 50 °C, diluted to a final volume of 400 mL with PBS, dialyzed against deionized water for seven days using 12–14 kDa membranes, lyophilized, and stored at −80 °C until use.

#### 2.1.3. Cell Encapsulation

**Figure 1:**
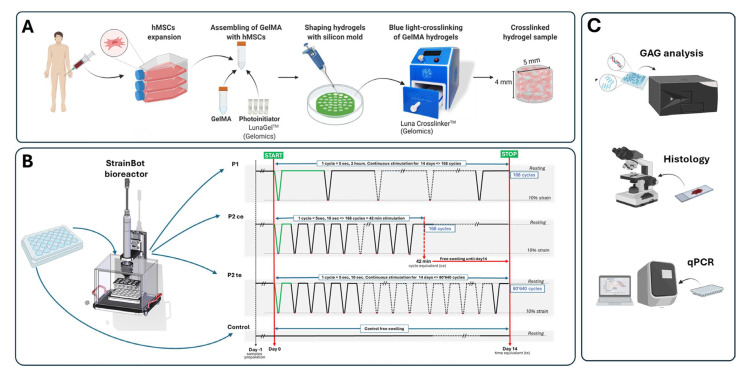
Study design. (**A**) Samples preparation workflow (**B**) Mechanical stimulation protocol using StrainBOT Bioreactor system with three distinct loading regimens: P1—5 s strain 2 h break (168 cycles over 14 days); P2ce—5 s strain 10 sec break (168 cycles over 42 min followed by free swelling until day 14); P2te—5 s strain 10 sec break (80′640 cycles over 14 days). In the Control group, free swelling without mechanical stimulation was run over 14 days. All groups maintained at 10% strain amplitude during compression phases, with resting periods between stimulation cycles. (**C**) Outcome measurements at day 14 included glycosaminoglycan (GAG) quantification using biochemical assays, histological analysis via standard histology and immunohistology staining techniques, and quantitative polymerase chain reaction (qPCR) for gene expression analysis.

MSCs were resuspended at a density of 40,000,000 cells per milliliter [25,27,28] of a 16% GelMa solution (*w*/*v* in PBS) and mixed with 0.15% *w*/*v* LAP photo-initiator (Gelomics Ltd., Brisbane, Australia) in equal volumes. The resulting 8% GelMa cell-laden solution was cast into silicon molds (Ø 5 × 4 mm) and cured for 8 min in the Luna Crosslinker (Gelomics) under visible light at an intensity of 9 mW/cm (Figure 1A).

### 2.2. Mechanical Stimulation

Cell-laden hydrogels were transferred into 24-well culture plates and maintained in DMEM High-Glucose supplemented with 50 μg/mL ascorbic acid 2-phosphate (Sigma–Aldrich), 100 nM dexamethasone (Sigma–Aldrich), 1% *v*/*v* Insuline-Transferrin-Selenium (ITS+) pre-mix (Corning), and 1% *v*/*v* non-essential amino acids (NEAA, Gibco). Constructs were then placed in the StrainBOT Bioreactor (RISystem, AG, Lanquart, Switzerland) for mechanical stimulation as described in Figure 1B. Medium was replaced twice a week.

In a previous study [25], we saw a positive effect of 14 days of continuous 10% deformation cycles (5 s strain-2 h break) on the hypertrophic chondrocyte differentiation of naïve MSCs when compared with the other groups, i.e., no strain day 0, day 14 no strain, and day 14 30% strain. Since no substantial difference was previously detected between 10% and 30% strain [25], in the present study, we focused on the application of 10% strain in three different protocols (Figure 1B) inspired by an earlier in vivo study from Hente and Perren [7].

Protocol 1 (P1): Samples were subjected to 5 s of strain followed by a 2 h pause (long-break cycles), repeated for 14 days, resulting in a total of 168 cycles.

Protocol 2 (P2): Samples were subjected to 5 s of strain followed by a 10 s pause (short-break cycles). To separately assess the influence of the (i) total number of cycles (168 vs. 80,640 cycles), (ii) pause duration (2 h vs. 10 s), and (iii) the overall stimulation duration (14 days vs. 42 min), two sub-protocols were implemented:

- P2ce (cycle equivalent): 168 cycles were condensed into 42 min (5 s strain + 10 s pause per cycle), after which samples were maintained in free swelling until day 14.
- P2te (time equivalent): Continuous stimulation for 14 days with 5 s strain + 10 s pause per cycle, resulting in a total of 80,640 cycles.

To confirm the specific effect of mechanical stimulation on the MSCs differentiation, a control group (control) was added, in which samples were cultured for 14 days in free-swelling conditions.

### 2.3. Cell Characterization

#### 2.3.1. DNA Content

DNA was quantified in one sample per condition per donor (n = 6) at days 1 and 14. Following overnight digestion in proteinase K (0.5 mg/mL) at 56 °C, 100 μL of the sample was added to 100 μL Quant-iT™ PicoGreen^®^ (Thermo-Fisher Scientific, Reinach, Switzerland) (fluorescent nucleic acid stain and incubated for 5 min. Fluorescence was subsequently measured using a TECAN Infinite 200 PRO M Plex reader (TECAN, Männedorf, Switzerland) (excitation 485 nm, emission 535 nm). Lambda DNA (100 μg/mL in TE) served as standard.

#### 2.3.2. Cell Morphology

Morphometric analyses were performed on ten times magnified H&E staining images (see Section 2.4.2) using Fiji/ImageJ-1.54 p analysis software. Images were manually threshold-adjusted, binarized, and segmented with sequential removal of noise, hole-filling, and watershed algorithms. Objects with areas of 150–3000 μm^2^ and circularity of 0.03–1.00 were counted as cells. Measurements were taken from 10 different fields of view across multiple sections and averaged.

#### 2.3.3. Gene Expression

Quantitative real-time PCR was conducted on samples from six donors (n = 6). Due to the high sensitivity of the technique, two samples per donor per condition (technical replicates) were analyzed. Results present the average of the duplicates for each donor. Total RNA was extracted as in [25]. Briefly, total RNA was extracted with 1 mL TriReagent^®^ and 5 µL polyacryl carrier (Molecular Research Center, Cincinnati, OH, USA), homogenized at 25 Hz for 6 min (TissueLyser, RETSCH, Haan, Germany). Phase separation was performed with 1-bromo-3-chloropropane (BCP; Sigma–Aldrich), and RNA was precipitated, washed thrice with 75% ethanol, air-dried, and resuspended in DEPC-treated water. RNA concentration and purity were determined using NanoDrop One (Thermo Fisher Scientific). One microgram of total RNA was used for cDNA synthesis using TaqMan^®^ Reverse Transcription reagents (Applied Biosystems, Thermo Fisher, Reinach, Switzerland) with random hexamer primers according to the manufacturer’s protocol. Quantitative PCR was conducted on a QuantStudio7 Flex instrument (Applied Biosystems, Thermo Fisher) using TaqMan^®^ gene assays or custom primer-probe sets (Microsynth, Balgach, Switzerland) as detailed in Table 1. Target genes included chondrogenic markers (*ACAN, COMP, SOX9*, and type II collagen (*COL2A1*)) and hypertrophic/late hypertrophic chondrocytes markers (type X Collagen (*COL10A1*), *MMP13, ALP, RUNX2*). *RPLP0* and *OAZ1* served as housekeeping genes for normalization. PCR amplification was performed using standard cycaling conditions: 10 min denaturation at 95 °C, followed by 40 cycles of 15 s at 95 °C and 1 min at 60 °C. Relative gene expression was assessed using the 2^−ΔΔCt^ method except for *Col2A1* and *ACAN*, for which only ΔCt could be calculated since not detected at day 0. ΔCt values were calculated relative to the geometric mean of housekeeping genes, and ΔΔCt values were calculated relative to the day 0 baseline control (average from six donors, normalized to 1).

### 2.4. Extracellular Matrix Characterization

#### 2.4.1. Paraffin Embedding and Sectioning

Samples (one per donor and per condition, n = 6) were fixed in 4% formalin (Formafix AG, Hittnau, Switzerland), dehydrated through a 30–70% ethanol series, and embedded in gelatin–agarose (Spin Tissue Processor STP 120-2, Microm, Thermo Fisher) followed by paraffin embedding (Tissue Embedding Center EC 350-1, Microm). Sections of 5 µm were cut using a Paraffin HM 355 S microtome (Microm).

#### 2.4.2. Histology Staining

Hematoxylin Eosin (H&E): After deparaffinization and rehydration, sections were washed in deionized water (dH_2_O) and subjected to Mayer’s hematoxylin staining (Sigma) for 10 min. Samples were then sequentially washed in lukewarm tap water, rinsed in dH_2_O, and then incubated in 1% Eosin working solution (comprising 1% Eosin stock with 0.1% glacial acetic acid) for 10 min. After aqueous washes, sections were dehydrated through a graded ethanol series (70% and 96% for 10 s each, absolute ethanol twice for 2 min) and cleared in xylene (≥5 min) before mounting with Eukitt medium (Sigma).

Safranin O Fast Green: Following deparaffinization and rehydration, sections were stained with Safranin O Fast Green (Fluka, Buch, Switzerland). Samples were rinsed with deionized water, stained with Weigert’s hematoxylin for 10 min (Sigma–Aldrich), and washed under running tap water for 10 min. Collagen was visualized after 6 min incubation of the sections in 0.02% Fast Green (Sigma–Aldrich), followed by a 1% acetic acid wash (Fluka). Proteoglycans were stained with 0.1% Safranin O for 15 min (Sigma–Aldrich), differentiated in 70% ethanol for 25 s, dehydrated through graded alcohols, and mounted using Eukitt (Sigma–Aldrich).

#### 2.4.3. Immunohistochemistry

Type II collagen (CIICI antibody, Developmental Studies Hybridoma Bank, 1:200) and type X collagen (X53, ThermoFisher, 1:100) extracellular matrix deposition was assessed by immunohistochemistry. Following deparaffinization and rehydration, sections were digested with 2 mg/mL hyaluronidase (Sigma) to unmask antigenic sites and blocked with 5% horse serum in PBS-Tween (Vector Laboratories, Newark, CA, USA). Samples were incubated overnight with primary antibodies at the specified dilutions, washed with PBS-Tween, and treated with biotinylated horse anti-mouse secondary antibody (Vectastain ABC kit, 1:200) for 30 min at room temperature. Detection was performed using the Vectastain Elite ABC kit with DAB (Vector Laboratories), and sections were mounted with ProLong Gold Antifade containing DAPI (Molecular Probes, Invitrogen, Thermo Fisher). Negative controls without primary antibodies were included, and imaging was carried out on an Olympus BX63 microscope (Evident Europe GmbH, Hamburg, Germany).

#### 2.4.4. Glycosaminoglycan (GAG) Deposition

GAG content in proteinase-K-digested samples and corresponding media was determined using the 1,9-dimethylmethylene blue (DMMB) assay (one sample per donor per condition, n = 6). Chondroitin 4-sulfate sodium salt from bovine trachea (Fluka) served as standard (maximum concentration 1.25 μg/well). Absorbance was measured at 530 nm using a TECAN microplate reader. DMMB solution was prepared according to Farndale et al. [29]. GAG values were normalized to the corresponding DNA content.

### 2.5. Statistical Analysis

All experiments were performed using cells from 6 different donors (n = 6 biological replicates). For gene expression, analyses were performed in duplicate for each of the six donors, and results are presented as the average of the two technical replicates. Results were analyzed for statistical differences using GraphPad Prism 10.1.2 (GraphPad Software, Boston, MA, USA) software. Upon normal distribution of data, a two-way Analysis of Variance (ANOVA) including Tukey’s multiple comparison test was applied. In other cases, the Kruskal–Wallis test with Dunn’s correction was used. *p*-values < 0.05 were considered significant.

## 3. Results

### 3.1. Samples Characterization

The chondrogenic differentiation potential of the cells used in this study was characterized by Safranin O Fast Green staining performed on free-swelling samples culture for 14 days in the presence of chondrogenic medium (containing 10 ng/mL TGFβ1) (Supplemental Figure S1).

**Figure 2:**
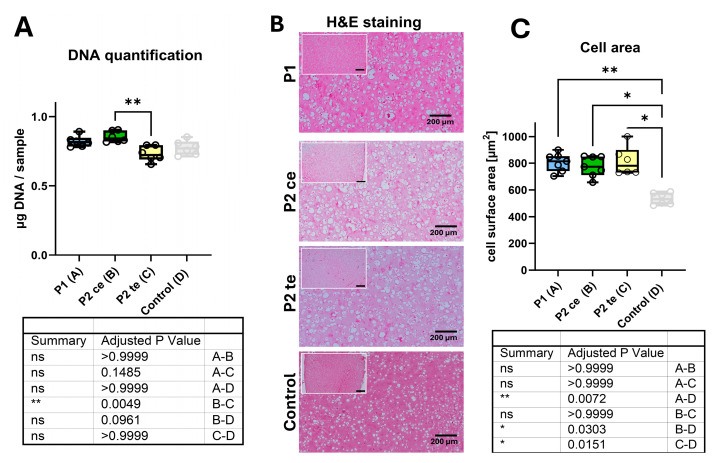
Samples characterization, comparison of DNA content, and cell morphology across experimental conditions. (**A**) DNA quantification showing significantly higher DNA content in P2ce (**B**) compared to P2te (**C**) (*p* = 0.0049). No significant differences were depicted among the other group comparisons. (**B**) Representative hematoxylin and eosin (H&E) stained tissue sections from each experimental group showing cellular morphology and density. Scale bar = 200 μm for the main image and 500 µm for the overview picture. (**C**) Cell size quantification was performed on ten fields of view of 2 sections from each tested condition. Results are presented as the average measurement for each individual donor. Quantitative analysis of cell surface area revealed no significant differences between the loaded groups. Cells in the Control group (free swelling) were significantly smaller compared to the three loaded groups. Data presented as box plots showing median, quartiles, and individual donor points (n = 6 per group). * *p* < 0.05, ** *p* < 0.01; ns, not significant.

DNA content analysis revealed some differences in cellularity between experimental groups (Figure 2A). P1 (14 days 168 cycles long break) and P2ce (168 cycles short break) groups demonstrated comparable DNA levels (0.82 ± 0.016 and 0.85 ± 0.016 μg DNA/sample, respectively), while P2te (14 days 80′640 cycles short break) showed a notable reduction in DNA content (0.732 ± 0.023 μg DNA/sample) being significant when compared to P2ce (*p* = 0.0049). No significant differences were observed between P1 and P2ce or between P2ce and P2te groups (*p* > 0.05). Differences quantified between the loaded samples and the free-swelling control group (0.773 ± 0.025 μg DNA/sample) were not statistically significant.

Histological analysis using hematoxylin and eosin staining revealed comparable overall characteristics across all conditions (Figure 2B). All groups, including control, displayed well-organized cellular distribution and evidence of extracellular matrix deposition. Despite this similarity, cells in the P1, P2ce, and P2te groups exhibited an enlarged morphology consistent with a hypertrophic chondrocyte phenotype, whereas cells in the control group appeared smaller. Morphometric analysis of cell surface area demonstrated no significant differences between the three loaded groups (Figure 2C), while in the free-swelling control group, the cells were significantly smaller, with the highest difference being between P1 and control (*p* = 0.0072). All three loaded protocols induced characteristic cellular enlargement consistent with hypertrophic chondrocyte morphology, with surface areas falling within the common range for hypertrophic cells (600–1000 μm^2^). Mean cell surface areas were 815 ± 45 μm^2^ for P1, 825 ± 75 μm^2^ for P2ce, and 795 ± 55 μm^2^ for P2te groups, respectively (*p* > 0.05 for all pairwise comparisons). The consistency of morphological changes across different loading protocols (i) confirms the effectiveness of mechanical loading in supporting hypertrophic differentiation compared to the control group, and (ii) supports the equivalent efficiency of brief early stimulation (P2ce) compared to extended loading regimens (P1 and P2te) in promoting it. Statistical comparison revealed no significant variations in cell size across the three loaded conditions (*p* > 0.9999).

### 3.2. Extra Cellular Matrix Characteristics

#### 3.2.1. Glycosaminoglycans (GAG) Synthesis Analysis

**Figure 3:**
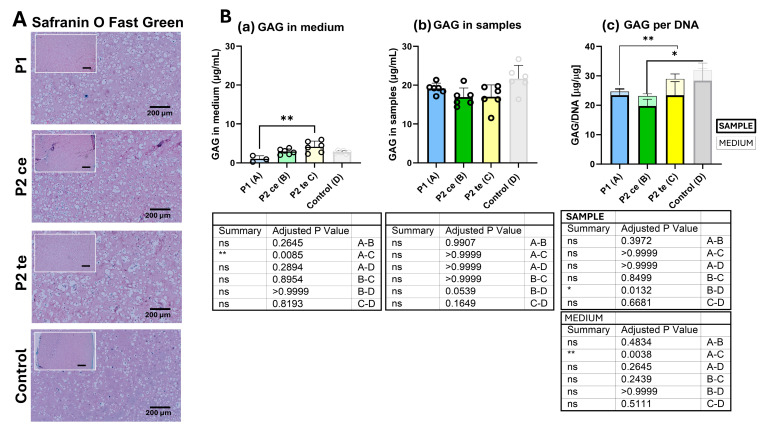
Glycosaminoglycan (GAG) production and distribution across experimental conditions. (**A**) Representative Safranin O Fast Green stained sample sections showing GAG distribution (red-ich staining) in the extracellular matrix. Scale bar = 200 μm for the main pictures and 500 µm for the overview images. (**B**) Quantitative analysis of GAG content in culture medium and tissue samples. Panel (**a**) shows GAG concentration in culture medium with P2te demonstrating significantly higher GAG release compared to P1 (*p* = 0.0085). Panel (**b**) displays total GAG content in samples, showing no significant differences between all experimental groups. Panel (**c**) presents GAG content normalized to DNA content. Data presented as mean ± SEM with individual donor points (n = 6 per group). Legend indicates sample (in bold character, thick outline, and sharp colors) vs. medium (normal character, thin outline, and faint colors) measurements. * *p* < 0.05, ** *p* < 0.01; ns, not significant.

Safranin O Fast Green histology staining first provided a qualitative evaluation of glycosaminoglycan deposition and distribution, as well as of collagenous matrix organization (Figure 3A). All tested groups exhibited low positive Safranin O staining (typically red), indicating a low glycosaminoglycan deposition throughout the constructs and in all conditions. Fast Green counterstaining (typically green/turquoise) revealed collagenous matrix distribution, with comparable overall staining across all conditions. The absence of clear red (GAG) or clear green/turquoise (collagenous matrix) coloration also indicates (i) a low GAG deposition, and (ii) a probable colocalization of the red Safranin O and the green Fast green staining in the samples. In addition, the coloration patterns demonstrated uniform matrix deposition with no obvious differences in glycosaminoglycan density or distribution between P1, P2ce, and P2te groups. Likewise, no apparent differences were depicted when compared to the free-swelling control group.

Biochemistry GAG analysis refined this observation. GAG synthesis was measured in samples of 6 donors for each tested group. GAG production was evaluated both in its released form in the surrounding cell-culture medium (Figure 3(Ba)) and its accumulation in the samples’ matrix (Figure 3(Bb)). Figure 3(Bc) shows the GAG quantification normalized to the related DNA content.

GAG accumulation in the culture medium (Figure 3(Ba)) differed significantly only between P1 and P2te. The P1 group showed minimal release (1.01 ± 0.70 μg/mL), whereas P2te exhibited the highest release (4.13 ± 0.14 μg/mL; *p* = 0.0085), significantly higher than in the P1 condition (*p* = 0.0085). The medium GAG levels for P2ce (2.88 ± 0.72 μg/mL) and free-swelling control (2.76 ± 0.38 μg/mL) did not differ significantly from either P1 or P2te.

GAG content within samples remained comparable across all groups (Figure 3(Bb)). P1, P2ce, and P2te groups demonstrated similar GAG accumulation (19.14 ± 1.38, 16.91 ± 2.30, and 17.01 ± 3.41 μg/mL, respectively) with no statistical differences reached. In the control group, slightly higher GAG was measured in the samples (21.67 ± 3.39 μg/mL), nearly reaching statistical significance when compared to P2ce (*p* = 0.0539).

Normalization of GAG content to DNA (Figure 3(Bc)) revealed comparable GAG/DNA ratios in P1 and P2te (23.37 ± 1.90 and 23.36 ± 4.62 μg/μg DNA, respectively). The P2ce group exhibited a lower ratio (19.76 ± 2.26 μg/μg DNA), although this reduction was not statistically significant when compared with P1 or P2te. When compared to the free-swelling control (28.33 ± 6.03 μg/μg DNA), only P2ce demonstrated a significant decrease (*p* = 0.0132) (thick bar). These findings indicate comparable per-cell GAG production capacity across most loading conditions and are consistent with the Safranin O/Fast Green staining results.

#### 3.2.2. Type II and Type X Collagen Immunostaining

**Figure 4:**
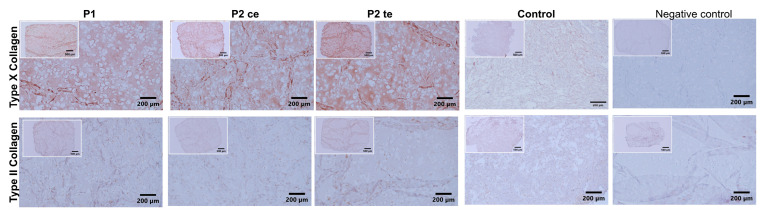
Immunohistochemical analysis of collagen types X and II expression across experimental conditions. Representative tissue sections showing collagen distribution patterns in experimental groups compared to the negative control. Upper panel: type X collagen immunostaining (brown) demonstrates positive staining in P1, P2ce, and P2te groups, with higher expression in P2ce and P2te conditions compared to P1 and notably lower staining in the free-swelling Control group. Lower panel: type II collagen immunostaining shows minimal to absent expression across all experimental groups. Negative control sections (left column) confirm antibody specificity with the absence of nonspecific staining. Scale bar = 200 μm for all images and 500 µm for the overview pictures.

Immunohistochemical analysis demonstrated distinct expression patterns of hypertrophic chondrocytes (type X Collagen) and chondrocyte (type II collagen)-associated markers between the loaded samples (P1, P2ce, and P2te) and free-swelling control (Figure 4). For both immunostainings, the antibody specificity was confirmed by the absence of nonspecific binding in the negative control sections. Strong positive staining was observed for the loaded constructs, namely P1, P2ce, and P2te conditions, where cells displayed pronounced type X collagen deposition along with a hypertrophic morphology. No noticeable differences were observed between the three groups. In contrast, the free-swelling control exhibited weak or absent staining, with only a faint background signal detected. A minimal positive immunostaining response was observed for type II collagen across the P1, P2ce, and P2te groups. The limited type II collagen detection indicated a reduced chondrocyte-specific matrix deposition, compatible with further cell differentiation towards hypertrophic chondrocytes. Here again, no apparent differences were observed between the three experimental conditions or the free-swelling control. The differential expression pattern between type X and type II collagen confirmed a predominantly hypertrophic chondrocyte phenotype in all loaded constructs.

#### 3.2.3. Gene Expression Analysis

**Figure 5:**
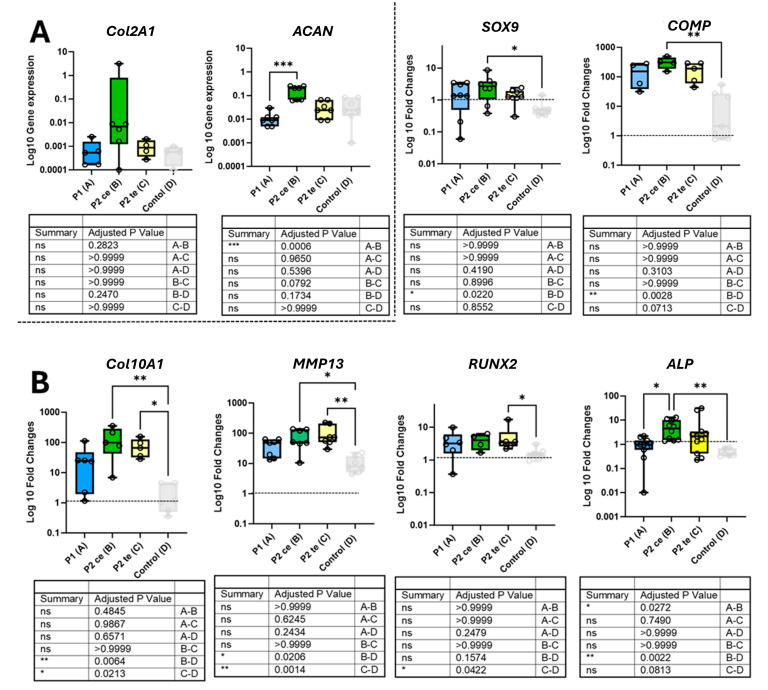
Gene expression analysis of chondrocytes and hypertrophic chondrocyte-related markers across experimental conditions. (**A**) Expression of chondrocyte-associated genes showing *Col2A1* (type II collagen) with increased expression in P2ce compared to the other groups. *ACAN* (aggrecan) demonstrated significantly higher expression in P2ce versus. *SOX9* expression was significantly increased in P2ce compared to Control. *COMP* showed significantly higher expression in P2ce versus free-swelling Control. (**B**) Expression of hypertrophic chondrocyte markers revealing *Col10A1* (type X collagen) with no significant variations between the three loaded groups, but significant upregulation in P2ce and in P2te compared to free swelling. *MMP13* was significantly elevated in P2ce and in P2te versus Control. *RUNX2* showed increased expression in P2te compared to Control. In both cases, no significant differences were detected between the three loaded groups. *ALP* demonstrated significant upregulation in P2ce versus P1 and P2te versus Control. Data presented as log_10_ fold-changes relative to baseline (dotted line at 1.0) except for *Col2A1* and *ACAN* expressed as log_10_ gene expression with individual donor points (average of technical replicates) overlaid on box plots showing median and quartiles (n = 6 per group). * *p* < 0.05, ** *p* < 0.01, *** *p* < 0.001; ns, not significant.

Figure 5 presents the gene expression and regulation of chondrocyte (Figure 5A) and hypertrophic chondrocytes (Figure 5B)-related genes. Except for *Col2A1* and *ACAN*, not detected at day 0 [27], all gene expression data were normalized to the day 0 unstrained baseline using the ΔΔCt method and presented as Log10 fold-changes. The baseline expression level is indicated by the dashed line.

Chondrocyte-related genes are shown in Figure 5A. *Col2A1* expression was low across all tested conditions, including the free-swelling control. While P2ce tended to increase *Col2A1* gene expression, no statistically significant differences were reached. *ACAN* demonstrated a significantly higher expression under P2ce compared to P1 (*p* = 0.0006), while no significant difference was observed between P1 and P2te (*p* = 0.4140) or comparing P2ce with P2te (*p* = 0.9389). For both *Col2A1* and *ACAN*, no significant differences were observed between any of the loaded groups and the free-swelling control group. *SOX9* expression showed no significant differences between the three loading protocols, despite a slight (non-significant) upregulation in P2ce samples. Similarly, *COMP* gene expression remained comparable across all three loading protocols with no significant inter-group differences. Although all loaded groups tended to exhibit higher *SOX9* and *COMP* gene expression compared to the free-swelling control, only P2ce induced a significant upregulation of *SOX9* (*p* = 0.022) and *COMP* (*p* = 0.0028). 

The investigation of hypertrophic chondrocyte-related genes (Figure 5B) revealed similar expression patterns across the three loading conditions. While P2ce showed a trend of higher *Col10A1* gene expression, no statistical differences were observed between the three loaded groups. Significant upregulation of *Col10A1* was observed in P2ce (*p* = 0.0064) and P2te (*p* = 0.0213) as compared to the control group. No statistical differences were observed for *MMP13* between P1, P2ce, and P2te, while P2ce or P2te induced a significant *MMP13* upregulation as compared to free-swelling control (*p* = 0.0206 and *p* = 0.0014, respectively). Following the same trend, *RUNX2* expression remained stable across all loaded groups, showing a general tendency toward higher regulation compared to the free-swelling control, with a significant difference observed between P2te and the control group (*p* = 0.0422). *Col10A*, *MMP13*, and *RUNX2* expression remained comparable across P1, P2ce, and P2te protocols with no significant inter-group variations, while being generally upregulated compared to the control. While P2ce and P2te showed a significantly upregulation of *Col10A1* and *MMP13* compared to control (*p* = 0.0064, *p* = 0.0213 for *Col10A1* and *p* = 0.0206 and *p* = 0.0014 for *MMP13*), *ALP* gene expression showed the only significant difference between the three loaded groups. P2ce demonstrated a significantly higher expression of ALP compared to P1 (*p* = 0.0272) and to the free-swelling control group (*p* = 0.0022), but not with P2te (*p* > 0.9999). P2te induced intermediate *ALP* levels without significant differences from either P1, P2ce or free-swelling control group (*p* = 0.0813).

## 4. Discussion

The success of bone-healing critically depends on the mechanical environment at the fracture site [1,11,30], and the link between mechanical conditions and fracture healing is well established. However, there is no consensus on the optimal stability and stimulation parameters leading to successful healing, as different studies report varying findings and recommendations [20,31,32]. These divergent opinions are not only due to methodological differences, but also, and mainly, to the complex interplay between biological and mechanical factors and the lack of knowledge at the cellular level [20,25,31].

Previous studies from our group showed that MSCs under 10% strain (5 s–2 h break, 14 days) developed hypertrophic chondrocyte characteristics including elevated type X collagen and *MMP13* expression [33,34,35,36,37], reduced GAG synthesis [34,38], and increased cell volume [34,39,40] compared to standard chondrogenic differentiation or to free-swelling condition in both chondrogenic and chondro-permissive conditions [25,41]. Likewise, cell viability, assessed via live-dead staining and apoptosis-related gene expression, was not affected in any of the tested conditions, nor did media and cells show chondrogenic differentiation potential when TGFβ was added to the medium. Building on the robust and reproducible results from our previous study [25], we reproduced the 10% strain condition in our protocol 1 and maintained the day 14 endpoint to ensure methodological consistency and facilitate direct comparison across studies. In the present study, we investigated how different mechanical stimulation protocols affect MSCs differentiation. In the context of secondary bone-healing, we focused on the effect of the number of cycles, the duration of mechanical stimulation, as well as the recovery period between cycles, on the hypertrophic chondrocyte differentiation of the cells. We examined the effect of three mechanical loading protocols on MSCs hypertrophic differentiation: P1 (long break, 168 cycles in 14 days) [25,41,42], P2ce (short break, 168 cycles in 42 min followed by free swelling), and P2te (short break, 80,640 cycles in 14 days). In the control group, samples were incubated in the same conditions but without mechanical stimulation (Figure 1B).

Our in vitro bioreactor-based findings provide key insights into the fundamental relationship between mechanical stimulation parameters and cellular responses.

### 4.1. Mechanical Stimulation Drives Hypertrophic Differentiation of Naïve MSCs

We previously showed that 10–30% strain induces a hypertrophic chondrocyte–like phenotype without exogenous chondrogenic factors, with 10% strain proving equally effective [25]. Accordingly, the present study focused on applying a 10% strain to confirm and extend these findings.

The P1 protocol (5 sec strain, 2-h break for 14 days, 168 cycles) in the present study directly replicates the optimal conditions identified in our initial work [25]. The upregulation of type X collagen (at both gene and protein level), *MMP13*, *RUNX2*, and *ALP*, markers of hypertrophic chondrocyte [35,38,43,44,45], along with reduced type II collagen and GAG deposition [38,46,47], confirms the robustness of the mechanical stimulation approach for inducing hypertrophic chondrocyte differentiation. This hypertrophic phenotype was also indicated by the low glycosaminoglycan production, with no statistical differences between all three loaded conditions. This aligns with previous work describing a decreasing GAG production along with higher *MMP13* production and type X collagen synthesis, along with hypertrophic chondrocyte differentiation in fracture callus, in osteoarthritis-related hypertrophy, or during development [33,48,49,50,51]. The inverse relationship between GAG synthesis and hypertrophic markers reflects the central shift in the cellular matrix production in preparation for vascular invasion and upcoming mineralization, characteristic of the endochondral ossification process [36,43,52,53]. This was supported by our morphological analyses showing that upon mechanical loading, cells adopted an enlarged and rounded morphology characteristic of hypertrophic chondrocytes [34,39,40,43]. Consistent with the hypertrophic differentiation observed under the P1 protocol, both P2ce and P2te protocols demonstrated comparable efficiency in driving hypertrophic differentiation of naïve MSCs.

Early studies on human MSCs pellet cultures in chondrogenic medium demonstrated gradual expression of type II and type X collagens. Johnstone et al. and Yoo et al. [28,54] reported type II collagen at day 7 of culture, followed by type X at day 14. Using MSC TGFβ-treated pellet cultures, Sekiya et al. [55] observed a peak of *MMP13* expression at day 7, accompanied by the expression of both type II and type X collagen, which continued to increase through day 21, while *MMP13* levels declined over time. In contrast, previous studies have reported a possible [36,50] early upregulation of hypertrophy marker type X collagen and *ACAN*, in some cases preceding type II collagen expression. Our findings and those of Jörimann et al. [25] (2024) show elevated type X collagen and *MMP13* with minimal type II collagen under mechanical stimulation at day 14, which could suggest a mechanical route from pre-chondrocyte phenotype to hypertrophy, even though an earlier peak of type II collagen at day 7 cannot be excluded [27]. In our model, mechanical stimulation also induced *RUNX2*, a key transcription factor for hypertrophic chondrocyte maturation [43,45,56,57], alongside type X collagen, further supporting mechanically driven hypertrophic differentiation.

### 4.2. Influence of Specific Mechanical Loading Protocols

Our comparison between protocol P1 (5 s strain-2 h break) and protocol P2 (5 s strain-10 s break) provides new insights into the impact of recovery periods between loading cycles. Despite the markedly different pause intervals, both protocols effectively induced hypertrophic differentiation via the mechanical induction and regulation of the intracellular signaling pathway [58]. In a mouse tibia fracture model, Gardner et al. showed efficient and timely bone-healing when a 9 s rest was inserted between loading cycles [15]. On the other hand, in a sheep experimental bone injury model (tilting wedge), Hente and Perren [7] showed a higher callus formation in their 2 h break group compared to 10 s. These large discrepancies can be due to many factors, such as the animal model, the bone injury type, or the mechanical loading regimen applied (i.e., number of cycles), further underlining the need for systemic investigation of the effect of mechanical loading in the context of endochondral bone-healing. In our in vitro study, no significant differences were observed between P1 (2 h break) and P2 (te and ce both 10 s break) regarding the cell-differentiation pattern.

However, comparing P2ce (168 cycles) and P2te (80,640 cycles), P2te samples displayed a significantly reduced DNA content relative to P2ce (0.85 ± 0.03 and 0.73 ± 0.05 μg/sample, respectively), suggesting that excessive mechanical loading may impair cell viability or proliferation. Beyond this reduction, no additional significant differences were detected in terms of cell differentiation, suggesting a possible plateau effect of mechanical loading. This latest observation is supported by previous work showing a plateau effect of mechanical loading [7,9,59,60,61]. Extended stimulation may induce a lower cellular response when the amount of signal exceeds the adaptive response threshold of the cells [58,62], supporting the idea of an optimal loading window [8,16,63].

This concept of plateau was not only observed regarding the number of cycles (P2ce versus P2te), but also while comparing the total duration of the mechanical loading (i.e., P1 168 cycles over 14 days versus P2ce 168 cycles in 42 min followed by 14 days free swelling). In this case, our data showed equivalent cellular response when samples were subjected to either a brief 42-min stimulation time followed by 14-day free swelling or permanent stimulation for 14 days. The efficacy of the early brief stimulation suggests the activation of a persistent mechanotransduction pathway maintaining a lasting cellular response even after the stimulation has stopped. This is supported by the findings of Yang et al., who showed that human MSCs possess a mechanical memory, involving the activation of *YAP/TAZ* and *RUNX2*, and that mechanical dosing beyond a threshold leads to irreversible activation of these pathways [64]. This aligns with Sittichokechaiwut et al.’s observation [65]. Despite their focus on mature osteoblasts rather than naïve MSCs, they showed that the mechanical loading effect persists beyond the stimulation period. In our study, the persistence of hypertrophic markers (type X collagen, *MMP13, RUNX2, ALP*) at day 14, in all three loaded protocols and despite only 42 min stimulation in P2ce, indicates that initial mechanical signals could trigger a self-sustaining differentiation cascade and further suggests that an early short mechanical boosting can initiate transcriptional programs that become autonomous [24,45]. Indeed, the increased expression of *RUNX2* in all three loading protocols compared to control (2.5, 2.4, and 3.5 fold increase for P1, P2ce, and P2te, respectively) could indicate a positive feedback loop maintaining R*UNX2*’s own expression and that of downstream hypertrophic genes [56,66]. This is supported by the work of Zheng et al., showing that *RUNX2* can self-regulate and maintain hypertrophic differentiation programs once activated [45]. Indeed, as described by Thompson, the activation of mechanosensitive ion channels, integrin signaling pathways, and transcriptional cascades remains active long after the initial mechanical trigger [58].

These mechanisms are particularly relevant for the P2ce group, where a brief 42-min stimulation produced effects comparable to the two longer protocols.

The comparable effectiveness of short (10 s) and long (2 h) pause intervals suggests that even brief recovery phases allow for effective mechanotransduction. This supports the concept of “mechanical priming,” where early signals can trigger transcriptional and structural programs that persist independently of continued stimulation [64,67,68]. Such a mechanism aligns with pre-clinical observations that early mobilization accelerates bone repair [8,16,18]. The persistence of hypertrophic markers over 14 days, even after only 42 min of stimulation in the P2ce protocol, strongly suggests that initial mechanical inputs are sufficient to initiate self-sustaining differentiation cascades.

### 4.3. Implications for Clinical Translation

Numerous studies have investigated the role of stability on bone fracture healing in experimental setups or clinical investigations, leading to the design and/or choice of appropriate implants or rehabilitation protocols [10,13,69]. However, although the effect of the mechanical environment has been studied in vivo at the tissue level, its effect at the cellular level has been less investigated. Despite many years of development and usage of bioreactors for the study of the mechanical effect at the cellular level, only very few bioreactor-based studies focus on the hypertrophic differentiation in the context of bone endochondral healing [70], even though the development of new implants would greatly benefit from a better knowledge of the mechanical environment at the cellular level. We believe that our approach can provide a framework that may translate into adapted rehabilitation protocols, emphasizing early, controlled mobilization rather than prolonged immobilization. Furthermore, identifying an optimal stimulation window opens opportunities for the design of smart implants and fixation devices delivering time-efficient mechanical loading during the critical early phase of healing [1,2,9].

### 4.4. Limitations and Future Directions

Several limitations of our study should be acknowledged. First, only female bone marrow-derived MSCs were used to maintain consistency with our previous study [25]. While donor age can affect chondrogenic potential [71,72], bone marrow source (e.g., vertebra, iliac crest) appears to have a greater influence on cell yield, proliferation, and differentiation [73]. The donor-specific differences observed in our study did not appear to be age-related, suggesting this limitation does not substantially affect data interpretation.

Second, our in vitro model, while sophisticated, cannot fully replicate the complex multicellular environment of healing bone tissue. Indeed, while this study demonstrated the differentiation of MSCs toward a hypertrophic chondrocyte phenotype—demonstrated by the sustained expression of key markers such as type X collagen (*Col10A1*) and *MMP13* even after brief stimulation—already after short term stimulation, the long-term functional implications of these findings remain to be elucidated. Future investigations should determine whether the mechanically induced hypertrophic phenotype promotes mineralization, supports appropriate matrix organization, and would ultimately enable successful transition to bone formation. In vivo validation of these processes will be essential to confirm their relevance for fracture healing. Elucidating the mechanotransduction pathways triggered by early stimulation could reveal therapeutic targets, particularly relevant in compromised healing.

## 5. Conclusions

Our study shows that brief early mechanical stimulation promotes hypertrophic chondrocyte differentiation of naïve MSCs, comparable to prolonged loading. The finding that 42 min of stimulation can elicit similar effects to 14 days of continuous loading supports the concept of mechanical priming, in which initial mechanical signals can initiate and sustain differentiation programs. While the duration of inter-cycle breaks influenced cellular responses, it did not fundamentally alter the differentiation outcome, and excessive cycle numbers did not confer additional benefits and may even compromise cell viability.

These observations carry potential implications for both basic science and clinical practice. At a cellular level, they support the idea that MSC mechano-transduction may operate through a threshold-based mechanism, where early activation of signaling pathways can induce sustained output. From a translational perspective, our data provides preliminary support for exploring more efficient rehabilitation strategies, with the possibility of reducing patient burden and healthcare costs.

## Figures and Tables

**Table 1:** List of assays ID (assay on demand) and primer—probe sequences (forward, reverse, and probe) used for RT-qPCR.

**Assay on demand**	Assay ID		
*OAZI*	Hs00427923_m1		
*ALP*	Hs00758162_m1		
*COL2A1* (type II collagen)	Hs00264051_m1		
*COMP*	Hs00164359_m1		
*SOX9*	Hs00165814_m1		
**Mycrosynth**	Forward	Reverse	Probe
*RPLP0*	5’-TGG GCA AGA ACA CCA TGA TG-3’	5’-CGG ATA TGA GGC AGC AGT TTC-3’	5’-AGG GCA CCT GGA AAA CAA CCC AGC-3’
*COL10A1* (type X collagen)	5’-ACG CTG AAC GAT ACC AAA TG-3’	5’-TGC TAT ACC TTT ACT CTT TAT GGT GTA-3’	5’-ACT ACC CAA CAC CAA GAC ACA GTT CTT CAT TCC-3’
*ACAN*	5’-AGT CCT CAA GCC TCC TGT ACT CA-3’	5’-CGG GAA GTG GCG GTA ACA-3’	5’CCG GAA TGG AAA CGT GAA TCA GAA TCA ACT-3’
*MMP13*	5’-CGG CCA CTC CTT AGG TCT TG-3’	5’-TTT TGC CGG TGT AGG TGT AGA TAG-3’	5’-CTC CAA GGA CCC TGG AGC ACT CAT GT-3’
*RUNX2*	5’-AGC AAG GTT CAA CGA TCT GAG AT-3’	5’TTT GTG AAG ACG GTT ATG GTC AA-3’	5’-TGA AAC TCT TGC CTC GTC CAC TCC G-3’

## Data Availability

Data can be shared individually upon reasonable request addressed to the corresponding author.

## Supplementary Materials

The following supporting information can be downloaded at: https://www.mdpi.com/article/10.3390/cells14221773/s1. Figure S1: Chondrogenic potential of bone marrow derived MSCs.
