# Supplementary material for: Transient Early Mechanical Loading Induces Hypertrophic Chondrocyte Differentiation of Human Mesenchymal Stromal Cells
Source: Cells. 2025 Nov 12;14(22):1773. doi: 10.3390/cells14221773 (PMC12651168; doi:10.3390/cells14221773)
Supplement: Supplementary file 1 [file cells-14-01773-s001.zip › cells-3923820-supplementary.pdf]

cells

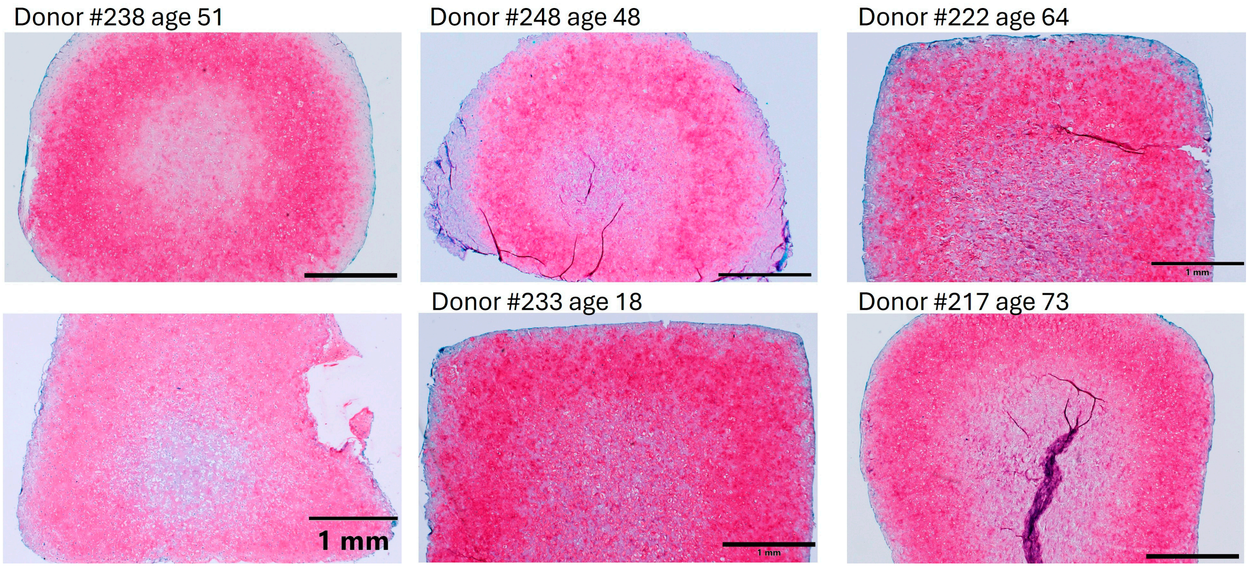

Figure S1. Chondrogenic potential of bone marrow derived MSCs from six donors. Safranin O/Fast Green staining confirmed the chondrogenic differentiation capacity of cells derived from the six donors used in this study. Histological staining was performed on thin paraffin-embedded sections of cell-laden GelMA hydrogels cultured for 14 days in chondrogenic medium. Sample identification numbers and corresponding donor ages are indicated on each image. Scale bar = 1 mm in all images.
